# Tartary Buckwheat in Human Nutrition

**DOI:** 10.3390/plants10040700

**Published:** 2021-04-05

**Authors:** Zlata Luthar, Aleksandra Golob, Mateja Germ, Blanka Vombergar, Ivan Kreft

**Affiliations:** 1 Biotechnical Faculty, University of Ljubljana, SI-1000 Ljubljana, Slovenia; zlata.luthar@bf.uni-lj.si (Z.L.); aleksandra.golob@bf.uni-lj.si (A.G.); mateja.germ@bf.uni-lj.si (M.G.); 2 The Education Centre Piramida Maribor, SI-2000 Maribor, Slovenia; blanka.vombergar@guest.arnes.si; 3 Nutrition Institute, Tržaška 40, SI-1000 Ljubljana, Slovenia

**Keywords:** Tartary buckwheat, retrograde starch, proteins, phenolic substances, flavonoids, anti-virus

## Abstract

Tartary buckwheat (*Fagopyrum tataricum* Gaertn.) originates in mountain areas of western China, and it is mainly cultivated in China, Bhutan, northern India, Nepal, and central Europe. Tartary buckwheat shows greater cold resistance than common buckwheat, and has traits for drought tolerance. Buckwheat can provide health benefits due to its contents of resistant starch, mineral elements, proteins, and in particular, phenolic substances, which prevent the effects of several chronic human diseases, including hypertension, obesity, cardiovascular diseases, and gallstone formation. The contents of the flavonoids rutin and quercetin are very variable among Tartary buckwheat samples from different origins and parts of the plants. Quercetin is formed after the degradation of rutin by the Tartary buckwheat enzyme rutinosidase, which mainly occurs after grain milling during mixing of the flour with water. High temperature treatments of wet Tartary buckwheat material prevent the conversion of rutin to quercetin.

## 1. Introduction

**Figure 1:**
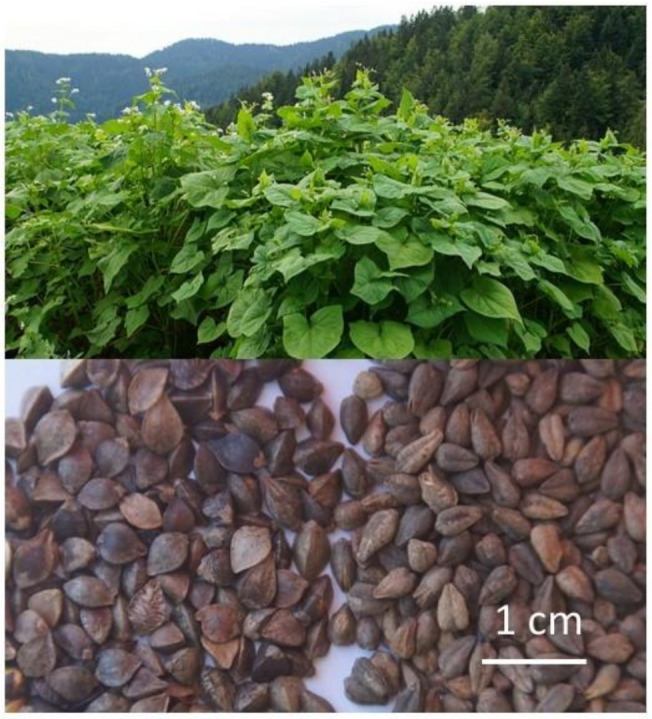
Tartary buckwheat (right, green flowering) and common buckwheat (to the left, white flowering) plants, and seeds.

Tartary buckwheat (*Fagopyrum tataricum* Gaertn.) originated in western China [1], and it is grown in mountain areas of China, Bhutan, northern India, and Nepal [2,3]. In the same countries in regions with less harsh climatic conditions, Tartary buckwheat is grown along with common buckwheat (Figure 1). In Europe, Tartary buckwheat is traditionally cultivated and widely used in Luxemburg, and in adjacent areas of Belgium and Germany, and it is also known in Slovenia and Italy [3,4,5]. Tartary buckwheat is also grown traditionally in Bosnia and Herzegovina as well as in a mixed crop with common buckwheat. Recently, it was reported that more than 100 hectares of Tartary buckwheat are grown annually in Värmland, Sweden [6].

Collection of Tartary buckwheat samples from Slovenian fields started in the late 1970s. At that time, it was cultivated by only a few farmers, and instead, in many buckwheat fields, it coexisted with common buckwheat, but as a weed. A pluriannual research project for the Luxembourgish Ministry of Agriculture has boosted research into, and cultivation and development of, Tartary buckwheat in Europe [3,4].

Common and Tartary buckwheat have different growth characteristics [7]. Tartary buckwheat is known to be resistant to the effects of cold weather, due to its epigenetic regulation by DNA methylation [8]. It is also more drought resistant than common buckwheat. Indeed, Tartary buckwheat has traits of drought tolerance, while common buckwheat has properties of drought avoidance [9].

The genus *Fagopyrum* includes 21 species [1,10]. Two of these, *Fagopyrum esculentum* and *F. tataricum*, are used in human nutrition, and the wild species *Fagopyrum cymosum* is used in traditional Chinese human and veterinary medicines. Wild relatives of cultivated buckwheat have spread through the mountain areas of south-western China [11], where recently a new self-compatible species was found and described as *Fagopyrum longistylum* by M. Zhou and Y. Tang [12]. *F. longistylum* and other wild buckwheat species can serve as donors of genes in the breeding of cultivated buckwheat species [13].

Tartary buckwheat includes a genotype called “rice-Tartary buckwheat”. Removal of the husk (husking) of this variant is easier in comparison to other Tartary buckwheat variants, as the husk is thinner and more brittle [14]. Comparative analysis of the transcriptomes of these two Tartary buckwheat genotypes has shown that 9250 genes are differentially expressed between them. These include differences in regulatory and structural genes that affect the chemical components of the cell wall. As Tartary buckwheat normally has a thicker and more robust husk than common buckwheat, this rice-Tartary buckwheat variant is important for easy husking to obtain groats.

Tartary buckwheat accessions reveal multiple domestication sites, as has been shown by sequencing of large numbers of Tartary buckwheat samples [12]. Intraspecific crosses that included Tartary buckwheat have led to several new hybrid species, among which the best known is probably *Fagopyrum giganteum* Krotov, which was originally defined by Krotov and Dranenko at the Ustymivska Experimental Station in Ukraine [15,16]. The wild species *Fagopyrum homotropicum* has been an important source for the development of self-pollination in cultured buckwheat species [17].

Buckwheat intake has protective effects against several chronic diseases, including hypertension, obesity, cardiovascular diseases, and gallstone formation [18]. These effects are mainly attributed to the resistant starch, protein, and phenolic substances in the buckwheat grain.

## 2. Resistant Starch

The amylose content of starch is the basis for the appearance of retrograde starch [19]. Amylose is the starting material to obtain resistant starch using various hydrothermal treatments [20]. Resistant starch is the part of starch that is not digested by human enzymes before entering the colon. The amount of resistant starch is affected by the composition of the starch, in terms of its high amylose content, and depending on ecological and genetic factors [21,22,23]. In Tartary buckwheat variety Xinong9920, starch peak viscosity was 2121 cP, and in the same conditions the peak viscosity of variety Xinong9940 was significantly less, namely 1928 cP [22]. In the experiment with phosphorus fertilization it was in Tartary buckwheat at 75 kg/ha P dose in starch 24.7% apparent amylose in total starch (27.0% in non-fertilized), and in another experiment 27.4% (28.6% in non-fertilized) in total amount of starch [23].

Fertilization of Tartary buckwheat with phosphate affects its growth, development and quality. Zhang et al. [23] showed that fertilization with different phosphorus (P) levels (from 15–135 kg/ha) affects the characteristics of starch from Tartary buckwheat. The increasing P content initially decreased and after that increased the apparent amylose content and mean diameter of the starch granules. These effects had impact on starch retrogradation, the process whereby following mixing of starch with hot water, the disaggregated amylose and amylopectin chains then undergo recombination upon cooling, to form a more ordered structure. The retrogradation rate of Tartary buckwheat starch pastes increased during the first 8 h after the start of cooling and then gradually stabilized. Increased amylose content promotes rapid retrogradation, and slow starch retrogradation is an effect of amylopectin molecules. Accordingly, starch from Tartary buckwheat exposed to high-P treatments has a high number of branched long-chains of amylopectin, because a high in situ concentration of long chains occurs during the process of reformation of the starch helices. Hydrothermal treatment of Tartary buckwheat grain starch enhances the levels of slowly digestible starch due to the retrograde starch [24].

Buckwheat grains have relatively small starch granules, with an amylose content of the grain starch higher than that of cereals [25]. As Tartary buckwheat has a high flavonoid content, which can interact with starch molecules, Tartary buckwheat can be used for the production of foods with a low glycemic index [22]. Increased resistant starch is obtained by cooking the buckwheat groats [25], and also by cold plasma treatment and quercetin complexation [26]. This digestion of resistant starch is part of our dietary fiber, and it acts as a prebiotic.

Different Tartary buckwheat varieties have different starch characteristics, and thus it is necessary to take this into account during the processing of buckwheat [22]. The buckwheat growing conditions, the availability of organic matter during buckwheat grain filling and ripening, and also the hereditary characteristics of the buckwheat, all have an important effect on the size of the starch granules and the starch amylose content. Hydrothermally processed buckwheat samples contained up to 4% retrograde starch, in comparison to untreated and dry-heated buckwheat, which contains only about 1% resistant starch, as dry matter [19,27,28].

Progress in research into common and Tartary starch grain size and shape was reviewed by [22]. The lower starch glycemic index and insulin index after heating has been attributed to the formation of amylase-resistant starch [29].

Lactic acid bacterial cultures and bifidobacteria can be used to prepare beverages from common buckwheat starch-rich products. Tartary buckwheat bread products are also made from fermented dough in Slovenia [30,31].

## 3. Protein

Buckwheat grain is considered a pseudocereal with high nutritional value because of its protein composition. Although buckwheat grain has a low protein content (common buckwheat, 10.6 g/100 g dry weight; Tartary buckwheat, 10.3 g/100 g dry weight), it has a balanced amino-acid composition, with high levels of essential amino acids, such as leucine and lysine (common buckwheat: 6.92, 5.84 g/100 g protein; Tartary buckwheat: 7.11, 6.18 g/100 g protein; respectively) [2]. The high content of protein, flavonoids and trace elements in certain buckwheat grain milling fractions suggests their use in special dietary products [32]. Buckwheat grain protein can also contain Se [33], which is an essential trace element in human nutrition.

Different hydrothermal treatments of buckwheat grain have been studied to determine the impact of polyphenol levels on protein digestibility [29]. In a rat model system, considerable interactions were seen between polyphenols and protein during the hydrothermal treatments. These interactions reduced the digestion of the buckwheat grain protein in the small and large intestines. However, microbial processes in the colon enhanced the digestibility of the protein in the hydrothermally processed buckwheat that was otherwise blocked by polyphenols [29]. The authors established that polyphenols that are naturally present in buckwheat husks lower the true digestibility of buckwheat grain protein, but do not adversely affect the biological value. As reported by Ikeda et al. [34], tannic acid and catechin have significant inhibitory effects on in vitro peptic and pancreatic digestion of buckwheat globulin. Ikeda et al. [34] and Ikeda and Kishida [35] studied the in vitro digestibility of buckwheat grain protein and the impact of secondary buckwheat metabolites. Evidence in the literature indicates that buckwheat grain protein can reduce cholesterol levels in serum by increasing fecal excretion of steroids, which is induced by binding of steroids to undigested protein. According to Ma and Xiong [36], digestion-resistant peptides are largely responsible for bile acid elimination. These effects are most probably connected with the limited digestibility of buckwheat grain protein.

As buckwheat grain does not contain gluten proteins, it is used for the preparation of foods for patients with celiac disease [37,38]. Although buckwheat allergy is not very common, allergic disorders associated with eating buckwheat-based foods have been reported [39,40,41]. The low molecular weight buckwheat grain proteins that are associated with such allergies are located in the grain embryo, and not the endosperm [42].

During the traditional hydrothermal preparation of buckwheat groats, there is migration of the substances from the grain pericarp into the groats [43,44]. In the processing of buckwheat grain for different food products, there are various interactions possible among the constituents, and especially during hydrothermal treatments.

Jin et al. [45] suggested that buckwheat grain can be treated to improve the protein digestibility and the bioactivity of common and Tartary buckwheat protein. In this way, buckwheat grain can be used as a plant-based protein source for improvement of the global protein supply.

## 4. Mineral Elements

Tartary buckwheat grain and grain products have high levels of mineral elements [3]. These levels depend on the milling process, with the highest levels of mineral elements in the bran; less is in the dark flour, and the least in the fine, light flour. Rb and Ag levels are higher in common buckwheat than in Tartary buckwheat, although the levels of other mineral elements (e.g., Se, Zn, Fe, Co, Ni, Sb, Cr, Sn) are higher in Tartary buckwheat. However, the levels of all of the elements studied are a lot higher in Tartary buckwheat leaf flour than in the grain or the milled products of the grain.

Tartary buckwheat leaf infusions contain decreasing levels (in order) of Zn, Cu, Cr, Ni, Pb, and Cd [46]. The Cr concentrations in infusions from the whole plant and from the grain bran and embryo were reported in this study to be in the range of 2.5 mg/kg to 3.2 mg/kg. In samples collected from commercial markets in China, the part of the plant used and the processing methods were seen to impact upon these metal concentrations in the products. For the content of Cr, the Tartary buckwheat for these infusions needs to be grown in a clean environment.

Using the Tartary buckwheat green parts or bran in infusions has indicated that in comparison to beverages from groats, these infusions contain higher concentration of Cd (0.5–1.2 mg/kg), Pb (0.3–0.4 mg/kg), Cu (5–8 mg/kg), and Zn (30–50 mg/kg) [46]. Should the plant leaves and grain husks be contaminated with soil particles, it is possible that polluted soil represents the direct source of the contamination of Tartary buckwheat with metals. On the other hand, metal ions can bind to flavonoids, which might facilitate the absorption of metals into Tartary buckwheat plants, and their allocation thus to the parts that are rich in flavonoids. According to Li et al. [46], the Pb levels in leaves are not related to the flavonoids content, or to the total phenolics content. Tartary buckwheat grain flour from Luxemburg, in Europe, had only 0.32 mg/kg Cr in the dark flour, and only 0.10 mg/kg Cr in the fine flour [3].

Common buckwheat can accumulate Al in its leaves, although this Al storage is not expressed in the grain. This appears to be because there is no Al transportation via the phloem, and Al is not mobile after its accumulation in leaves. The accumulated Al in older leaves appears to originate from the roots, and therefore, green parts of buckwheat plants can be used to remove Al from the soil [47,48,49].

Buckwheat is known to be suitable for biofortification with Se while it can accumulate substantial amount of Se. As such, it can be a source of Se for the human diet [33,50]. Salicylic acid can increase the Se levels in plant tissues [51]. Of note, patients with severe COVID-19 infection have vitamin D and Se deficiencies. Indeed, as Se appears to enhance the cytotoxic effector cells, Se deficiency is a possible risk factor for COVID-19 mortality [52].

## 5. Phenolic Substances

Yu et al. [53] compared the rutin and quercetin contents across 44 Tartary buckwheat grain and sprout samples from China, Nepal, Bhutan, India, Japan, Pakistan, and Slovenia. These were very variable for these different origins (Table 1). The samples from Nepal had the highest concentrations of rutin in the grain (13.3 g/kg) and sprouts (54.4 g/kg). For quercetin, the sprouts contained 10–90-fold that seen in the grain [53]. Therefore, Tartary buckwheat sprouts have great potential for production of flavonoids and for functional foods that are rich in flavonoids.

According to Klykov et al. [54], in samples from the far east of Russia, common buckwheat grain contained 0.1% rutin by weight, Tartary buckwheat 2.4%, and cymosum buckwheat 1.1%. At full flowering, the aboveground parts of common buckwheat had 3.1% to 3.8% rutin, which produced 92 kg to 121 kg rutin/ha; the same for Tartary buckwheat indicated 4.1% to 4.4% rutin, for 107 kg to 129 kg rutin/ha, and for cymosum buckwheat, ~4.1% rutin for ~83 kg rutin/ha [54]. Of note, Tartary buckwheat with a dark grain cover contains more rutin than Tartary buckwheat varieties with other grain colors [55]. Additionally, ozone levels in the atmosphere can affect the biosynthesis of phenolic substances in Tartary buckwheat [56,57,58].

**Figure 2:**
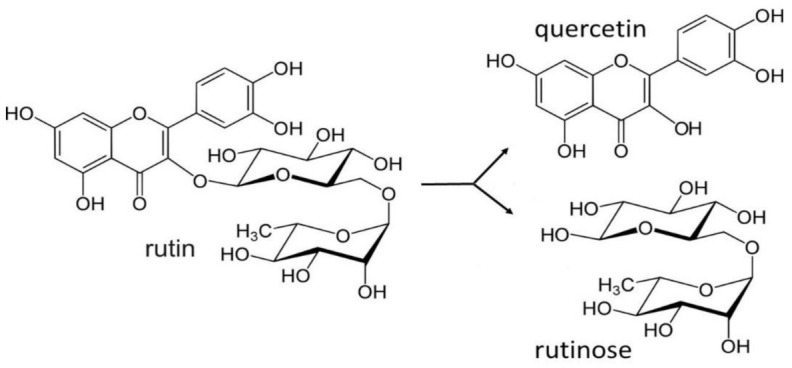
Rutin transformation to quercetin and rutinose.

Milling of the grain of Tartary buckwheat and mixing the flour with water results in the formation of quercetin, as a degradation product of rutin by rutinosidase (Figure 2) [59,60,61,62,63]. However, superheated steam or saturated steam can be used to inactivate the rutin-degrading enzymes in buckwheat flour in less than 90 s. In contrast, under far infrared drying, these rutin-degrading enzymes persist at 150 ℃ for 40 min [64].

Ingested quercetin can cross the blood–brain barrier and accumulate in the brain tissue [65]. Indeed, important bioactivities have been established for quercetin and its derivatives not just in blood vessels, muscle, and the gastrointestinal system, but also in the brain. Quercetin and other phenolics have been isolated from stool samples of people who had eaten food rich in phenolic substances [65]. The presence of phenolic substances in the colon can reduce the virus loads in the stools.

In Tartary buckwheat, quercetin complexation with starch molecules has an impact on the in vitro digestibility of the starch and the appearance of resistant starch, thus altering the physicochemical properties of the Tartary buckwheat starch [66]. The effects of this quercetin–polyphenol complexation indicate that food products based on Tartary buckwheat will show lower digestibility. Indeed, the quercetin in Tartary buckwheat can reduce body weight, serum triacylglycerols, and low-density lipoprotein. In rats, a diet with 0.1% quercetin was shown to have significant effects towards lowering low-density lipoprotein concentrations in serum, with no such effects on high-density lipoprotein. Tartary buckwheat has also been shown to prevent increases in body weight and fat deposition during high-fat intake in rats, although on the other hand, this was reported to protect against hepatic stenosis [67]. A buckwheat diet can also reduce insulin and ameliorate glucose intolerance in humans [19].

Rat experiments with common buckwheat have further suggested the complexity of the impact of the gut microbiota. Indeed, Peng et al. [67] suggested that the link between weight gain and the gut microbiota is very complex, with the need for further studies here.

Interestingly, it has been shown that rutin-enriched Tartary buckwheat flour extracts provide better flavonoid oral absorption, with the phenolic substances in the blood detectable for longer than with standard rutin, and even longer than for a native Tartary buckwheat grain flour extract [45]. Rutin is in the most part bound to other grain substances and structures. Indeed, extraction of rutin from untreated Tartary buckwheat grain flour showed 0.57 g rutin/100 g flour, while autoclaving resulted in 3.03 g/100 g flour, boiling resulted in 2.97 g rutin/100 g flour, and steaming resulted in 2.50 g/100 g flour [45,68]. Dzah et al. [68] also studied solid–liquid extraction conditions for Tartary buckwheat, where they indicated that the extraction of phenolic compounds from Tartary buckwheat flour can be performed at < 65 ℃.

The rutin and quercetin in Tartary buckwheat grain have an impact upon the physicochemical properties of the starch after cooking. The aging enthalpy of retrograde starch is lowered, and the viscosity of Tartary buckwheat starch and paste is increased. Starch–phenolics binding is stronger than that of the complex of starch and iodine. Starch is gelatinized and retrograde, and the morphology is affected by quercetin and rutin [69].

Among the phenolics, some resveratrol has been reported for flours of common buckwheat grain [70]. Nemcova et al. [71] reported from 1.0 mg/kg to 1.7 mg/kg *trans*-resveratrol for common buckwheat grain, while for Tartary buckwheat grain, there was ~3.5 mg/kg *trans*-resveratrol. Li et al. [72] reported that the resveratrol in Tartary buckwheat bran does not show any detectable resveratrol in a bound form.

Chen et al. [73] suggested a new three-solvent mix for efficient and comprehensive extraction of phenolics from Tartary buckwheat: acetone, ethyl acetate, and ethanol. This method is valuable for evaluation of the Tartary buckwheat functional properties. Extraction of rutin from buckwheat samples is more effective with around 70% ethanol, instead of more concentrated extractions [74]. As rutin and other phenolic substances can be bound to different compounds and grain structures, effective extraction can take several hours [30,31,59].

Buckwheat phenolic compounds can inhibit fungal development due to the phenolic hydrophobic interactions with cell membranes [75]. This effect is important for the antifungal properties of sourdoughs. Lactic acid bacteria can split flavonoid glycosides to flavonoid aglycones and a sugar, and can further metabolize aglycones. The resulting metabolites, which include lactic acid and other organic acids, also serve to increase the antifungal activity of buckwheat sourdough. This might explain the prolonged shelf life of Tartary buckwheat sourdough bakery products [75].

The slow digestion properties of starch were studied by Luo et al. [76], following ethanol extract of Tartary buckwheat. The slow digestibility of this starch appeared to be due to the impact of phenolic substances on the starch. In their in vivo experiments, mice showed reduced postprandial glycemic responses. These data of Luo et al. [76] for Tartary buckwheat grain and glycemic responses were similar to those obtained earlier in common buckwheat [19].

Phenolic compounds are often transformed in the gut before their absorption. The gut microbiota is important in this process [77]. Large-sized dietary phenolics are poorly absorbable, while small-sized products of microbial conversion are more easily absorbed in the colon.

Wieslander et al. [78,79] performed a comprehensive double-blind crossover study with 62 adult female participants, who additional to their normal diet, consumed either 359.7 mg rutin per day (high rutin diet; as Tartary buckwheat cookies), or 16.5 mg rutin per day (low rutin diet; as common buckwheat cookies). After two weeks, the groups changed their type of cookies (and hence rutin intake levels) for the following two weeks. Serum levels of myeloperoxidase (as an inflammation marker) were reduced significantly in the women who changed from low to high rutin in the diet (week 2 vs. week 4: reduction of 55.4 μg/mL; *p* < 0.02) [78]. The higher rutin in the Tartary buckwheat cookies was also related with strongly improved fatigue symptoms in comparison to the baseline, according to the reduction in a visual analogue rating scale (32 vs. 22; *p* ˂ 0.01). For total serum cholesterol levels, these were significantly reduced from baseline to four weeks for the combination of the data for low and high rutin regardless of the order of intake (5.31 vs. 4.59 mmol/L; *p* < 0.001). The authors reported that this indicated that the cholesterol lowering effect was not related to the content of rutin in the cookies, but to other constituents of the buckwheats as well. Vogrinčič et al. [80] reported that flavonoids are important in the antigenotoxic effects of Tartary buckwheat, although other buckwheat metabolites have also important effects.

Cyclitols (also known as D-chiro-inositols) have also been reported for Tartary buckwheat grain (0.18–0.20%) [81]. The synthesis of cyclitols is triggered by environmental parameters like salt stress and drought, and they can function as cryoprotectants. Accumulation of cyclitol metabolites is directly connected with abiotic stress factors. The environmental conditions of plants thus have an impact on the regulation of metabolic pathways for synthesis and accumulation of cyclitols [82]. These compounds are known to have anti-diabetic, anti-inflammatory, and other bioactivities in humans, as reviewed by Ratiu et al. [82].

Non-invasive methods for distinguishing between Tartary and common buckwheat samples based on flavonol accumulation in the green parts of the plants have been suggested [83]. Here, p-anisic acid in buckwheat leaves indicates Tartary buckwheat, rather than common buckwheat.

Consumption of Tartary buckwheat infusions is a tradition that is popular in China, and recently its consumption has spread also to Japan and Europe [84]. It was reported for a mouse model that herb extracts in combination with Tartary buckwheat grain infusion can reduce blood glucose levels, and lower serum triglycerides, total cholesterol, and high-density lipoprotein-cholesterol levels.

Tartary buckwheat grain malt is also used in the preparation of drinks and cookies. The malt is rich in orientin, vitexin, rutin, and quercetin, although the flavonoid levels in cookies made with Tartary buckwheat grain are lower than expected in terms of the amounts of raw Tartary buckwheat grain, soaked or germinated grain, or malt. Interestingly, the levels of these flavonoids in Tartary buckwheat malt were higher than in the intact, soaked, or germinated Tartary buckwheat products [37,85]. In the raw Tartary buckwheat it was 2.2 mg/g d.w. of rutin, and in whole Tartary buckwheat malt 3.7 mg/g d.w. of rutin [37].

Li et al. [72] reported antiproliferative effects of phenolic Tartary buckwheat extracts on human breast cancer cells in an in vitro cell model. These data remain to be confirmed by in vivo experiments.

In hydrothermally treated buckwheat, during the soaking of the grain, rutin moves from the bran fraction into the endosperm, which results in higher amounts of rutin in the flour [86]. Similar migration of smaller molecules from husk to groats during hydrothermal treatment was shown earlier for common buckwheat [43].

**Figure 3:**
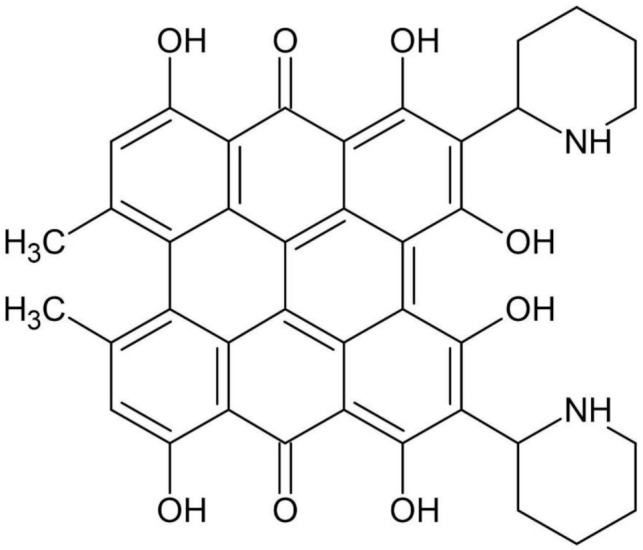
Fagopyrin.

The anthraquinone content in Tartary buckwheat has been studied in relation to the color of the grain husk [87]. The fagopyrin (Figure 3) levels are at their highest during seed germination, and light is important for the transformation of protofagopyrins into fagopyrins, as increased fagopyrin levels have been shown to accompany increased light conditions [88,89]. The consumption of green parts of buckwheat plants can cause fagopyrism, which involves photosensitization with serous exudate, skin irritation, and edema [88,89]. In fungi, fagopyrin is involved in the regulation of mycelial growth and morphology, and in pathogenicity [90].

**Figure 4:**
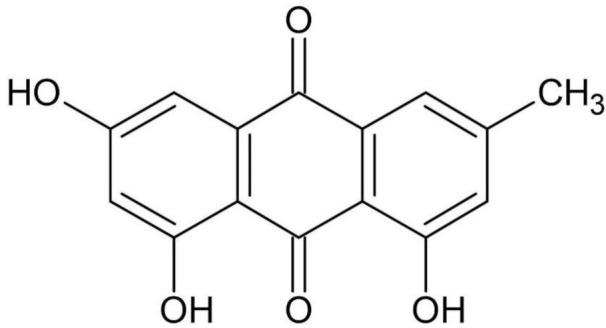
Emodin.

Emodin (Figure 4) has been reported for the bran and leaves of Tartary buckwheat [91]. It appears to be a precursor in the synthesis of hypericin, and of Tartary buckwheat fagopyrin [92]. Of importance here, emodin isolated from Tartary buckwheat grain has been shown to dock into all three active sites of the RNA-binding domain of the nucleocapsid phosphoprotein of SARS-CoV-2 [91,92,93,94].

Quercetin has potential therapeutic effects against acute kidney injury, and for treatment of impaired renal function. Tartary buckwheat grain extracts are rich in such flavonoids, and these can alleviate ethanol-induced liver injury in rats [87]. Rutin also protects type 2 diabetic mice against liver injury [95]. The same function is seen for Tartary buckwheat grain extracts, through inhibition of mitochondrial cell death [96,97]. In vivo pharmacokinetics have also provided support for the administration of Tartary buckwheat extracts to prevent alcoholic liver disease in humans [98].

Rutin is reported to also be effective in wound healing and in hyperglycemic rats, as it can reduce oxidative stress and inflammatory responses, to thus reduce the risk of ulcer formation [99]. Rutin induces activities of glutathione peroxidase, which protect the testis of adult rats against the effects of ethanol [100]. Tartary buckwheat flavonoids improve vascular sensitivity and show antihypertensive effects in spontaneously hypertensive rats [101].

Salicylaldehyde is the most characteristic compound of common buckwheat, and it has not been found in Tartary buckwheat. The aroma of Tartary buckwheat significantly differs from the aroma of common buckwheat; indeed, as salicylaldehyde is a volatile compound, it can be used as a marker for detection of contamination of Tartary buckwheat with common buckwheat [43,44]. Tartary buckwheat also contains naphthalene [44].

## 6. Conclusions

Foods made from the grain of Tartary buckwheat have shown preventive effects against several chronic diseases, including obesity, cardiovascular diseases, gallstone formation, and hypertension. The effects are mainly attributed to the resistant starch, protein and phenolic substances in the grain, and to the interactions among these constituents. Polyphenols have an impact on protein digestibility after hydrothermal treatment. Their interaction reduces the digestion of protein through the small and large intestines. Microbial processes in the colon enhance the digestibility of the grain protein and starch, which are otherwise blocked by polyphenols in hydrothermally processed buckwheat. Among the polyphenols, fagopyrin appears to pose a health threat when the green parts of the plants are consumed, and especially in summer by people with a light skin color. Consuming buckwheat grain and grain products has been shown to be safe.

Buckwheat protein can reduce serum cholesterol levels through increased fecal excretion of steroids, which is induced by the binding of steroids to undigested protein. Digestion-resistant peptides are largely responsible for bile acid elimination. As buckwheat does not contain the gluten proteins, it is used as food for people with celiac disease. The balanced amino-acid composition of buckwheat proteins represents an important source of dietary protein for people who maintain vegetarian or vegan diets. An increase in digestion-resistant starch is obtained by cooking buckwheat groats, and by cold plasma treatments and quercetin complexation, which all result in modifications to the Tartary buckwheat grain starch. Resistant starch acts as a dietary fiber, and has a function as a prebiotic. Tartary buckwheat is a crop that is traditionally used as a food in mountain areas in Asia and Europe. Based on its origin, Tartary buckwheat is a low input plant. Due to its content of flavonoids and other phenolic substances, Tartary buckwheat is resistant to plant diseases, pests, and damage by UV-B radiation. This makes Tartary buckwheat feasible to be grown as an organic and ecological crop, with little need for addition of artificial fertilizers, or of chemical treatments.

Last but not least, Tartary buckwheat reminds many consumers of the “good old days”, whereby Tartary buckwheat dishes have been rising in popularity, especially among food quality-conscious people in Asia and Europe. This provides the possibility now to develop new, more “modern”, food products based on old culinary traditions, with re-evaluation through contemporary scientific knowledge of the quality and potential of Tartary buckwheat.

## Figures and Tables

**Table 1:** Results on concentration of rutin in Tartary buckwheat samples (in dry matter, HPLC 1200 series Agilent, column YMC-pack OSD-AC18, 4.6 mm ID × 250 mm, S-5 μm, YMC Co., LTD., Japan), adapted from Yu et al. [53].

Location	Range of Rutin Concentration in Grain (mg/100 g)	Range of Rutin Concentration in Sprouts (mg/100 g)
Bhutan (*n* = 3)	753.8–911.5	3039.3–3160.2
China (*n* = 28)	364.2–1247.5	328.8–5214
India (*n* = 2)	304.5–487.5	1542–1822.7
Japan (*n* = 2)	769.1–902.4	3177.2–3825.7
Nepal (*n* = 5)	583–1326.5	3313–5440.4
Pakistan (*n* = 2)	675.2–901.3	2716.1–4088.6
Slovenia (*n* = 2)	625.4–798.6	3382.6–3603.8

## Data Availability

Not applicable.

## References

[1] Zhang K., He M., Fan Y., Zhao H., Gao B., Yang K., Li F., Tang Y., Gao Q., Lin T. (2021). Resequencing of global Tartary buckwheat accessions reveals multiple domestication events and key loci associated with agronomic traits. Genome Biol..

[2] Bonafaccia G., Marocchini M., Kreft I. (2003). Composition and technological properties of the flour and bran from common and tartary buckwheat. Food Chem..

[3] Bonafaccia G., Gambelli L., Fabjan N., Kreft I. (2003). Trace elements in flour and bran from common and tartary buckwheat. Food Chem..

[4] Zewen C., Kreft I., Chang K.J., Choi Y.S., Park C.H. (2003). Buckwheat without borders. Ethnobotany of Buckwheat.

[5] Giupponi L., Borgonovo G., Panseri S., Giorgi A. (2019). Multidisciplinary study of a little known landrace of *Fagopyrum tataricum* Gaertn. of Valtellina (Italian Alps). Genet. Resour. Crop Evol..

[6] Kreft I. (2020). Grenko Seme Tatarske Ajde.

[7] Kasajima S., Yoshimaru I., Itoh H. (2019). Differentiation and growth of a growing point until the stage of flower bud appearance in leading common buckwheat and Tartary buckwheat varieties in northern Japan. Fagopyrum.

[8] Song Y., Jia Z., Hou Y., Ma X., Li L., Jin X., An L. (2020). Roles of DNA methylation in cold priming in Tartary buckwheat. Front. Plant Sci..

[9] Aubert L., Decamps C., Jacquemin G., Quinet M. (2021). Comparison of plant morphology, yield and nutritional quality of *Fagopyrum esculentum* and *Fagopyrum tataricum* grown under field conditions in Belgium. Plants.

[10] Kumari A., Kumar Chaudhary H. (2020). Nutraceutical crop buckwheat: A concealed wealth in the lap of Himalayas. Crit. Rev. Biotechnol..

[11] Ohnishi O. (2020). The origin of cultivated buckwheat in Mankang district of the Sanjiang area of Eastern Tibet and its diffusion to India and the Himalayan hills. Folia Biol. Geol..

[12] Zhang K., Fan Y., Weng W., Tang Y., Zhou M. (2021). *Fagopyrum longistylum* (Poligonaceae), a new species from Sichuan, China. Phytotaxa.

[13] Woo S.H., Roy S.K., Kwon S.J., Cho S.W., Sarker K., Lee M.S., Chung K.Y., Kim H.H., Zhou M., Kreft I., Woo S.H., Chrungoo N., Wieslander G. (2016). Concepts, Prospects, and Potentiality in Buckwheat (*Fagopyrum eculentum* Moench): A research perspective. Molecular Breeding and Nutritional Aspects of Buckwheat.

[14] Li H.Y., Wu C.X., Lv Q.Y., Shi T.X., Chen Q.J., Chen Q.F. (2020). Comparative cellular, physiological and transcriptome analyses reveal the potential easy dehulling mechanism of rice-tartary buckwheat (*Fagopyrum tataricum*). BMC Plant Biol..

[15] Tryhub O., Burdyga V., Kharchenko Y., Havrylyanchyk R. (2018). Formation of buckwheat genepool collection in Ukraine and directions of its usage. Fagopyrum.

[16] Tryhub O. (2019). Research results of local buckwheat varieties and forms of Ukrainian origin. Fagopyrum.

[17] Campbell C.G., Nagano M. (2020). Buckwheat breeding. Past, present and future. Folia Biol. Geol..

[18] Wieslander G. (2020). Buckwheat in human health—A medical review. Folia Biol. Geol..

[19] Škrabanja V., Liljeberg Elmståhl H.G.M., Kreft I., Björck I.M.E. (2001). Nutritional properties of starch in buckwheat products: Studies in vitro and in vivo. J. Agric. Food Chem..

[20] Chou C., Wu M., Nurtama B., Jenshinn Lin J. (2010). Manufacture of resistant starch by different physical modifications and storage times. J. Food Agric. Environ..

[21] Chrungoo N., Dohtdong L., Chettry U., Zhou M., Kreft I., Woo S.H., Chrungoo N., Wieslander G. (2016). Diversity in seed storage proteins and their genes in buckwheat. Molecular Breeding and Nutritional Aspects of Buckwheat.

[22] Gao J., Kreft I., Chao G., Wang Y., Liu W., Wang L., Wang P., Gao X., Feng B. (2016). Tartary buckwheat (*Fagopyrum tataricum* Gaertn.) starch, a side product in functional food production, as a potential source of retrograded starch. Food Chem..

[23] Zhang W., Yang Q., Xia M., Bai W., Wang P., Gao X., Gong X., Feng B., Gao L., Zhou M. (2020). Effects of phosphate fertiliser on the physicochemical properties of Tartary buckwheat (*Fagopyrum tataricum* (L.) Gaertn.) starch. Food Chem..

[24] Xiao Y., Liu H., Wei T., Shen J., Wang M. (2017). Differences in physicochemical properties and in vitro digestibility between tartary buckwheat flour and starch modified by heat-moisture treatment. LWT Food Sci. Technol..

[25] Škrabanja V., Kreft I. (1998). Resistant starch formation following autoclaving of buckwheat (*Fagopyrum esculentum* Moench) groats. An In Vitro study. J. Agric. Food Chem..

[26] Gao S., Liu H., Sun L., Cao J., Yang J., Lu M., Wang M. (2021). Rheological, thermal and in vitro digestibility properties on complex of plasma modified Tartary buckwheat starches with quercetin. Food Hydrocoll..

[27] Škrabanja V., Laerke H., Kreft I. (1998). Effects of hydrothermal processing of buckwheat (*Fagopyrum esculentum* Moench) groats on starch enzymatic availability in vitro and in vivo in rats. J. Cereal Sci..

[28] Škrabanja V., Kreft I., Zhou M., Woo S.H., Chrungoo N., Wieslander G. (2016). Nutritional value of buckwheat proteins and starch. Molecular Breeding and Nutritional Aspects of Buckwheat.

[29] Škrabanja V., Lærke H.N., Kreft I. (2000). Protein-polyphenol interactions and in vivo digestibility of buckwheat groat proteins. Pflügers Arch..

[30] Lukšič L., Bonafaccia G., Timoracka M., Vollmannova A., Trček J., Nyambe T.K., Melini V., Acquistucci R., Germ M., Kreft I. (2016). Rutin and quercetin transformation during preparation of buckwheat sourdough bread. J. Cereal Sci..

[31] Lukšič L., Árvay J., Vollmannová A., Tóth T., Škrabanja V., Trček J., Germ M., Kreft I. (2016). Hydrothermal treatment of Tartary buckwheat grain hinders the transformation of rutin to quercetin. J. Cereal Sci..

[32] Škrabanja V., Kreft I., Golob T., Modic M., Ikeda S., Ikeda K., Kreft S., Bonafaccia G., Knapp M., Košmelj K. (2004). Nutrient content in buckwheat milling fractions. Cereal Chem..

[33] Golob A., Gadžo D., Stibilj V., Djikić M., Gavrić T., Kreft I., Germ M. (2016). Sulphur interferes with selenium accumulation in Tartary buckwheat plants. Plant Physiol. Biochem..

[34] Ikeda K., Oku M., Kusano T., Yasumoto K. (1986). Inhibitory potency of plant antinutrients towards the In Vitro digestibility of buckwheat protein. J. Food Sci..

[35] Ikeda K., Kishida M. (1993). Digestibility of proteins in buckwheat seed. Fagopyrum.

[36] Ma Y.Y., Xiong Y.L. (2009). Antioxidant and bile acid binding activity of buckwheat protein in vitro digests. J. Agric. Food Chem..

[37] Molinari R., Costantini L., Timperio A.M., Lelli V., Bonafaccia F., Bonafaccia G., Merendino N. (2018). Tartary buckwheat malt as ingredient of gluten-free cookies. J. Cereal Sci..

[38] Giménez-Bastida J.A., Zielinski H. (2015). Buckwheat as a functional food and its effects on health. J. Agric. Food Chem..

[39] Urisu A., Kondo Y., Morita Y., Yagi E., Tsuruta M., Yasaki T., Yamada K., Kuzuya H., Suzuki M., Titani K. (1995). Isolation and characterization of major allergen in buckwheat seed. Current Advances in Buckwheat Research.

[40] Wieslander G., Norbäck D. (2001). Buckwheat allergy. Allergy.

[41] Matsumoto R., Fujino K., Nagata Y., Hashiguchi S., Ito Y., Aihara Y., Takahashi Y., Maeda K., Sugimura K. (2004). Molecular characterization of a 10-kDa buckwheat molecule reactive to allergic patients’ IgE. Allergy.

[42] Ličen M., Kreft I. (2005). Buckwheat (*Fagopyrum esculentum* Moench) low molecular weight seed proteins are restricted to the embryo and are not detectable in the endosperm. Plant Physiol. Biochem..

[43] Janeš D., Prosen H., Kreft I., Kreft S. (2010). Aroma compounds in buckwheat (*Fagopyrum esculentum* Moench) groats, flour, bran, and husk. Cereal Chem..

[44] Janeš D., Prosen H., Kreft S. (2012). Identification and quantification of aroma compounds of tartary buckwheat (*Fagopyrum tataricum* Gaertn.) and some of its milling fractions. J. Food Sci..

[45] Jin J., Ohanenye I.C., Udenigwe C.C. (2020). Buckwheat proteins: Functionality, safety, bioactivity, and prospects as alternative plant-based proteins in the food industry. Crit. Rev. Food Sci. Nutr..

[46] Li Z., Li Z., Huang Y., Jiang Y., Liu Y., Wen W., Li H., Shao J., Wang C., Zhu X. (2020). Antioxidant capacity, metal contents, and their health risk assessment of tartary buckwheat teas. ACS Omega.

[47] Shen R., Ma J.F. (2001). Distribution and mobility of aluminium in an Al-accumulating plant, *Fagopyrum esculentum* Moench. J. Exp. Bot..

[48] Ma J.F., Zheng S.J., Matsumoto H., Hiradate S. (1997). Detoxifying aluminium with buckwheat. Nature.

[49] Ma J.F., Hiradate S. (2000). Form of aluminium for uptake and translocation in buckwheat (*Fagopyrum esculentum* Moench). Planta.

[50] Golob A., Stibilj V., Kreft I., Vogel-Mikuš K., Gaberscik A., Germ M. (2018). Selenium treatment alters the effects of UV radiation on chemical and production parameters in hybrid buckwheat. Acta Agric. Scand. Sect. B Plant Soil Sci..

[51] Kowalska I., Smoleń S., Czernicka M., Halka M., Kęska K., Pitala J. (2020). Effect of selenium form and salicylic acid on the accumulation of selenium speciation forms in hydroponically grown lettuce. Agriculture.

[52] Bae M., Kim H. (2020). The role of vitamin C, vitamin D, and selenium in immune system against COVID-19. Molecules.

[53] Yu J.H., Kwon S.J., Choi J.Y., Ju Y.H., Roy S.K., Lee D.G., Park C.H., Woo S.H. (2019). Variation of rutin and quercetin contents in Tartary buckwheat germplasm. Fagopyrum.

[54] Klykov A., Chaikina E., Anisimov M., Borovaya S., Barsukova E. (2020). Rutin content in buckwheat (*Fagopyrum esculentum* Moench, *F.*
*tataricum* (L.) Gaertn. and *F.*
*cymosum* Meissn.) growth in the far east of Russia. Folia Biol. Geol..

[55] Choi Y.M., Yoon H., Lee S., Hyun D.Y., Lee M.C., Oh S., Rauf M. (2021). Characterization of agro-morphological traits of Tartary buckwheat germplasm under spring cultivation and analysis of health-related primary bioactive components in seeds by HPLC method. J. Plant Biol..

[56] Sun W., Ma Z., Liu M. (2020). Cytochrome P450 family: Genome-wide identification provides insights into the rutin synthesis pathway in Tartary buckwheat and the improvement of agricultural product quality. Int. J. Biol. Macromol..

[57] Li X., Wu Z., Xiao S., Wang A., Hua X., Yu Q., Liu Y., Peng L., Yang Y., Wang J. (2020). Characterization of abscisic acid (ABA) receptors and analysis of genes that regulate rutin biosynthesis in response to ABA in *Fagopyrum tataricum*. Plant Physiol. Biochem..

[58] Jeon J., Baek S.A., Kim N.S., Sathasivam R., Park J.S., Kim J.K., Park S.U. (2020). Elevated ozone levels affect metabolites and related biosynthetic genes in Tartary buckwheat. J. Agric. Food Chem..

[59] Germ M., Arvay J., Vollmannova A., Toth T., Golob A., Luthar Z., Kreft I. (2019). The temperature threshold for the transformation of rutin to quercetin in Tartary buckwheat dough. Food Chem..

[60] Suzuki T., Morishita T., Takigawa S., Noda T., Ishiguro K. (2015). Characterization of rutin-rich bread made with ‘Manten-Kirari’, a trace-rutinosidase variety of Tartary buckwheat (*Fagopyrum tataricum* Gaertn.). Food Sci. Technol. Res..

[61] Suzuki T., Noda T., Morishita T., Ishiguro K., Otsuka S., Brunori A. (2020). Present status and future perspectives of breeding for buckwheat quality. Breed. Sci..

[62] Yasuda T., Nakagawa H. (1994). Purification and characterization of the rutin-degrading enzymes in Tartary buckwheat seeds. Phytochemistry.

[63] Fujita K., Yoshihashi T. (2019). Heat-treatment of Tartary buckwheat (*Fagopyrum tataricum* Gaertn.) provides dehulled and gelatinized product with denatured rutinosidase. Food Sci. Technol. Res..

[64] Wu X., Fu G., Li R., Li Y., Dong B., Liu C. (2020). Effect of thermal processing for rutin preservation on the properties of phenolics & starch in Tartary buckwheat achenes. Int. J. Biol. Macromol..

[65] Kawabata K., Mukai R., Ishisaka A. (2015). Quercetin and related polyphenols: New insights and implications for their bioactivity and bioavailability. Food Funct..

[66] Li Y., Gao S., Ji X., Liu H., Liu N., Yang J., Lu M., Han L., Wang M. (2020). Evaluation studies on effects of quercetin with different concentrations on the physicochemical properties and in vitro digestibility of Tartary buckwheat starch. Int. J. Biol. Macromol..

[67] Peng L., Zhang Q., Zhang Y., Yao Z., Song P., Wei L., Zhao G., Yan Z. (2019). Effect of tartary buckwheat, rutin, and quercetin on lipid metabolism in rats during high dietary fat intake. Food Sci. Nutr..

[68] Dzah C.S., Duan Y., Zhang H., Boateng N.A.S., Ma H. (2020). Ultrasound-induced lipid peroxidation: Effects on phenol content and extraction kinetics and antioxidant activity of Tartary buckwheat (*Fagopyrum tataricum*) water extract. Food Biosci..

[69] He C., Zhang Z., Liu H., Gao J., Li Y., Wang M. (2017). Effect of rutin and quercetin on the physicochemical properties of Tartary buckwheat starch. Starch Starke.

[70] Qian J.Y., Mayer D., Kuhn M. (1999). Flavonoids in fine buckwheat (*Fagopyrum esculentum* Monch) flour and their free radical scavenging activities. Dtsch. Lebensm. Rundsch..

[71] Nemcová L., Zima J., Barek J., Janovská D. (2011). Determination of resveratrol in grains, hulls and leaves of common and tartary buckwheat by HPLC with electrochemical detection at carbon paste electrode. Food Chem..

[72] Li F., Zhang X., Li Y., Lu K., Yin R., Ming J. (2017). Phenolics extracted from tartary (*Fagopyrum tartaricum* L. Gaertn.) buckwheat bran exhibit antioxidant activity, and an antiproliferative effect on human breast cancer MDA-MB-231 cells through the p38/MAP kinase pathway. Food Funct..

[73] Chen Y., Qin L., Wen A., Mazhar M., Wang H., Zhu Y. (2020). Three-solvent extracting method comprehensively evaluates phenolics profile and antioxidant activities of Tartary buckwheat. J. Food Process. Preserv..

[74] Kreft I., Fabjan N., Yasumoto K. (2006). Rutin content in buckwheat (*Fagopyrum esculentum* Moench) food materials and products. Food Chem..

[75] Koval D., Plockova M., Kyselka J., Skřivan P., Sluková M., Horáčková S. (2020). Buckwheat secondary metabolites: Potential antifungal agents. J. Agric. Food Chem..

[76] Luo K., Zhou X., Zhang G. (2019). The impact of Tartary buckwheat extract on the nutritional property of starch in a whole grain context. J. Cereal Sci..

[77] Selma M.V., Espin J.C., Tomas-Barberan F.A. (2009). Interaction between phenolics and gut microbiota: Role in human health. J. Agric. Food Chem..

[78] Wieslander G., Fabjan N., Vogrincic M., Kreft I., Janson C., Spetz-Nyström U., Vombergar B., Tagesson C., Leanderson P., Norbäck D. (2011). Eating buckwheat cookies is associated with the reduction in serum levels of myeloperoxidase and cholesterol: A double blind crossover study in day-care centre staffs. Tohoku J. Exp. Med..

[79] Wieslander G., Fabjan N., Vogrinčič M., Kreft I., Vombergar B., Norbäck D. (2012). Effects of common and Tartary buckwheat consumption on mucosal symptoms, headache and tiredness: A double-blind crossover intervention study. J. Food Agric. Environ..

[80] Vogrinčič M., Kreft I., Filipič M., Žegura B. (2013). Antigenotoxic effect of tartary (*Fagopyrum tataricum*) and common (*Fagopyrum esculentum*) buckwheat flour. J. Med. Food..

[81] Yang N., Ren G. (2008). Application of near-infrared reflectance spectroscopy to the evaluation of rutin and D-chiro-inositol contents in tartary buckwheat. J. Agric. Food Chem..

[82] Ratiu I.A., Al-Suod H., Ligor M., Monedeiro F., Buszewski B. (2020). Effects of growth conditions and cultivability on the content of cyclitols in *Medicago sativa*. Int. J. Environm. Sci. Technol..

[83] Sytar O., Bruckova1 K., Plotnitskaya A., Zivcak1 M., Brestic M. (2019). Non-destructive methodology in comparative physiology of buckwheat genotypes within the different origin. Fagopyrum.

[84] Qin P., Wu L., Yao Y., Ren G. (2013). Changes in phytochemical compositions, antioxidant and α-glucosidase inhibitory activities during the processing of tartary buckwheat tea. Food Res. Int..

[85] Zhao X., Li C., Jiang Y., Wang M., Wang B., Xiao L., Xu X., Chai D., Dong L. (2020). Metabolite fngerprinting of buckwheat in the malting process. J. Food Meas. Charact..

[86] Oh M., Oh I., Jeong S., Lee S. (2019). Optical, rheological, thermal, and microstructural elucidation of rutin enrichment in Tartary buckwheat flour by hydrothermal treatments. Food Chem..

[87] Yang W., Su Y., Dong G., Qian G., Shi Y., Mi Y., Zhang Y., Xue J., Du W., Shi T. (2020). Liquid chromatography–mass spectrometry-based metabolomics analysis of flavonoids and anthraquinones in *Fagopyrum tataricum* L. Gaertn. (tartary buckwheat) seeds to trace morphological variations. Food Chem..

[88] Kim J., Hwang K.T. (2020). Fagopyrins in different parts of common buckwheat (*Fagopyrum esculentum*) and Tartary buckwheat (*F. tataricum*) during growth. J. Food Compos. Anal..

[89] Huda M.N., Lu S., Jahan T., Ding M., Jha R., Zhang K., Zhang W., Georgiev M.I., Park S.U., Zhou M. (2021). Treasure from garden: Bioactive compounds of buckwheat. Food Chem..

[90] Zambounis A., Sytar O., Valasiadis D., Hilioti Z. (2020). Effect of photosensitisers on growth and morphology of *Phytophthora citrophthora* coupled with leaf bioassays in pear seedlings. Plant Protect. Sci..

[91] Peng L.X., Wang J.B., Hu L.X., Zhao J.L., Xiang D.B., Zou L., Zhao G. (2013). Rapid and simple method for the determination of emodin in Tartary buckwheat (*Fagopyrum tataricum*) by high-performance liquid chromatography coupled to a diode array detector. J. Agric. Food Chem..

[92] Rolta R., Yadav R., Salaria D., Sourirajan A., Dev K. (2020). In silico screening of hundred phytocompounds of ten medicinal plants as potential inhibitors of nucleocapsid phosphoprotein of Covid-19: An approach to prevent virus assembly. J. Biomol. Struct. Dyn..

[93] Robson B. (2020). Computers and viral diseases. Preliminary bioinformatics studies on the design of a synthetic vaccine and a preventative peptidomimetic antagonist against the Sars-Cov-2 (2019-Ncov, Covid-19) coronavirus. Comput. Biol. Med..

[94] Subbaiyan A., Ravichandran K., Singh S.V., Sankar M., Thomas P., Dhama K., Malik Y.S., Singh R.K., Chaudhuri P. (2020). In silico molecular docking analysis targeting Sars-Cov-2 spike protein and selected herbal constituents. J. Pure Appl. Microbiol..

[95] Liang W., Zhang D., Kang J., Meng X., Yang J., Yang L., Xuea N., Gao Q., Han S., Gou X. (2018). Protective effects of rutin on liver injury in type 2 diabetic db/db mice. Biomed. Pharmacother..

[96] Yang Q., Luo C., Zhang X., Liu Y., Wang Z., Cacciamani P., Shi J., Cui Y., Wang C., Sinha B. (2020). Tartary buckwheat extract alleviates alcohol-induced acute and chronic liver injuries through the inhibition of oxidative stress and mitochondrial cell death pathway. Am. J. Transl. Res..

[97] Jin H.R., Lee S., Choi S.J. (2020). Pharmacokinetics and protective effects of Tartary buckwheat flour extracts against ethanol-induced liver injury in rats. Antioxidants.

[98] Liu Y., Gan J., Liu W., Zhang X., Xu J., Wu Y., Yang Y., Si L., Li G., Huang J. (2019). Pharmacokinetics and novel metabolite identification of Tartary buckwheat extracts in beagle dogs following co-administration with ethanol. Pharmaceutics.

[99] Chen L.Y., Huang C.N., Liao C.K., Chang H.M., Kuan Y.H., Tseng T.J., Yen K.J., Yang K.L., Lin H.C. (2020). Effects of rutin on wound healing in hyperglycemic rats. Antioxidants.

[100] Abarikwu S.O., Olufemi P.D., Lawrence C.J., Wekere F.C., Ochulor A.C., Barikuma A.M. (2016). Rutin, an antioxidant flavonoid, induces glutathione and glutathione peroxidase activities to protect against ethanol effects in cadmium-induced oxidative stress in the testis of adult rats. Andrologia.

[101] Hou Z., Hu Y., Yang X., Chen W. (2017). Antihypertensive effects of Tartary buckwheat flavonoids by improvement of vascular insulin sensitivity in spontaneously hypertensive rats. Food Funct..

